# Phylogenetic Position of Hungarian Grey Cattle Breed Based on Total-Representation Sample

**DOI:** 10.3390/ani15091186

**Published:** 2025-04-22

**Authors:** Ákos Maróti-Agóts, Zsombor Wagenhoffer, Csilla Józsa, Endre Kaltenecker, Balázs Kemény, Kristóf Csurgay, Benedek Zsigmond, Irene Cardinali, Hovirag Lancioni, András Gáspárdy

**Affiliations:** 1 Institute of Animal Breeding Nutrition and Laboratory Animal Science, University of Veterinary Medicine Budapest, István u. 2, 1078 Budapest, Hungary; 2 National Food Chain Safety Office, Animal Health Diagnostic, Cseri út 18, 7400 Kaposvár, Hungary; 3 Hungarian Grey Cattle Breeders Association, Lőportár u. 16, 1134 Budapest, Hungary; 4 All-in Vet, Budai út 76, 2030 Érd, Hungary; 5 Department of Mongolian and Inner Asian Studies, Eötvös Loránd University, Múzeum Krt. 4, 1088 Budapest, Hungary; 6 Department of Chemistry, Biology and Biotechnology, University of Perugia, Via Elce di Sotto, 06123 Perugia, Italyhovirag.lancioni@unipg.it (H.L.)

**Keywords:** Hungarian Grey cattle, total-representation sample, founder sampling, phylogeny, mitochondrial control region, taurine mitochondrial haplogroup

## Abstract

The Hungarian Grey (HG) cattle breed was almost extinct after WW2; only 200 cows and six bulls survived. Despite the historical significance of the HG, no comprehensive genomic analysis has been conducted to clarify its genetic diversity and evolutionary history. Previous studies have relied on random or limited pedigree sampling, lacking a fully representative dataset determining genetic and conservation status. Here, the founder sampling of 110 individuals and the analysis of their mitochondrial DNA (mtDNA) sequence variation aim to investigate the phylogenetic placement of the breed using, for the first time, a fully representative sample. All identified haplogroups belong to the taurine T macro-haplogroup, with a predominance of T3 (89.1%), followed by T2 (4.5%), T1 (3.6%), and T1′2′3 (2.7%). The phylogenetic analysis confirms the absence of ancient haplogroups derived from European aurochs, suggesting a purely taurine origin for the HG breed. The high haplotype diversity (Hd = 0.94) and the genetic similarity to other Podolian breeds, particularly Maremmana cattle, indicate a preserved genetic background despite centuries of selective breeding. The lack of intensive crossbreeding practices has maintained the original beef production purpose of the breed, distinguishing it from the crossbred Podolian cattle used for dual-purpose or dairy production.

## Simple Summary

The Hungarian Grey cattle breed was almost extinct after WW2, with only 200 cows and six bulls surviving. Therefore, it was possible to generate a complete representative sample for phylogenetic analysis of the mitochondrial DNA (mtDNA) inherited along maternal lineages. Our results show a high genetic variability and testify that the breed conserves the original taurine (T) mtDNA sequences and does not carry genetic traces of hybridization with European aurochs. This is presumably because this breed has never been used for anything other than extensive beef production and, therefore, was not hybridized. This suggests that the *original* Podolian breeds not used for intensive production are genetically distinct from other *intensive* Podolian breeds.

## 1. Introduction

There are many theories about the origin of the Podolian cattle breeds, including the Hungarian Grey (HG) [1,2]. Interestingly, about the phenotypically very similar Maremmana and HG breeds, there is a widespread theory that these Podolian cattle arrived in their current countries with the Etruscans and the Hungarian conquerors [2,3].

The first scientific description of Podolian cattle was given in 1841 by Moritz Wagner, whose color lithography of a dark grey bull was published in a book [4]. Charles Darwin, on the other hand, wrote in 1868 about pale “Hungarian cattle”: “In other parts of Europe there are several distinct races, such as the pale-coloured Hungarian cattle, with their light and free step, and their enormous horns sometimes measuring above five feet from tip to tip” [5]. This sentence refers to light-colored cattle.

In a recent article, Podolian cattle are divided into two groups according to their coat color, and HG cattle are also mentioned here as members of the darker grey group [2]. This is interesting because at the end of August in Hortobágy, only pigmented bulls are grey, and cows are off-white. However, this is measured with an objective colorimeter device [6].

The two most common inland theories about the origin of the HG are that they arrived in Hungary with the Hungarian conquerors or were domesticated from the aurochs (*Bos primigenius* Bojanus, 1827) stock of the Carpathian Basin aurochs by the settlers [7].

Archaeological excavations have shown that the settlers arrived in the Carpathian Basin predominantly with small ruminants, not cattle. No cattle of the same body proportions, possibly with long horns and such a body size, have been found in Hungary during this period [8]. What is certain, however, is that the source of this widespread theory is Árpád Feszty’s well-known romantic panorama painting “*Arrival of the Hungarians*” (1893).

Jankovich suggested that, in medieval Hungary, the occupation ‘*venatores bubalinorum*’, known from 13th-century documents, was associated with domestication [9]. He interpreted this expression as meaning ‘Bubalus calves’, i.e., catchers of the offspring of aurochs. At this time, however, aurochs bones were already rare at archaeological sites in Hungary [7].

The presence of aurochs in Asia [10] and in Europe, in the Carpathian Basin, on the other hand, is a subject that has been the subject of much genetic research. The publication by Achilli and colleagues (2008) [11] was the first to clarify the haplogrouping of ancestral and modern taurine sequences based on the results of whole-mitogenome sequencing after the genetic results of the millennium [12,13,14,15]. In the 2020s, an intensive archaeogenetic study of ancestral bones through the genetic analysis of different archaeological sites has elucidated the common history of European ancestral and modern taurine bovine ecosystems [16,17].

The emergence of the HG in Hungary around the 9th–14th centuries and its relationship with the Italian Maremmana breed is not yet clear. Still, by the 16th century, the Hungarian large-horned cattle ‘*magnus cornuotes boves Hungaricos*’ [18] was an important export good [19]. It has also been suggested that the long horns were visible from far away, a trademark of the HG, which at the time was a meat of exceptional quality and there was even a statue of it in Fleiscbrücke (1599) in Nuremberg [20].

The HG stock, which was dwindling in numbers, was almost extinct after the Second World War for political reasons. The bulls of the severe bottleneck herd (200 cows and six bulls) were rescued from the slaughterhouse by Professor Imre Bodó in Hortobágy in 1961 [1]. Today, the status of the HG is stable in Hungary, with more than 10,000 registered cows.

More comprehensive mitochondrial studies have shown that aurochs haplogroups are present in European cattle breeds [11]. Thus, it became clear that European taurine cattle hybridized with aurochs, as confirmed by the P, Q haplotypes. Therefore, the later arriving taurine cattle mixed with the local ancestral aurochs, and these haplotypes are still found today due to intensive crossbreeding practices. A PQT mitochondrial haplogroup has also been found in the Podolia breed group, suggesting aurochs hybridization [21].

To date, there has been no comprehensive genomic analysis on the HG breed, and information on its molecular diversity, genetic history, and conservation status is still lacking. Mitochondrial genetic studies focusing on HG phylogeny have been predominantly random, rarely based on pedigree sampling. Thus, a representation of the sample has, therefore, not been declared so far.

This study aimed to determine the phylogeny of the HG cow families based on the mitochondrial control region (CR) sequences of a fully representative sample. The HG sample in this research was totally representative.

## 2. Materials and Methods

The “founder sampling” was carried out very accurately, using all the breeding documents to reconstruct the bottleneck population [22]. The visualization capabilities of the yEd Network v3.23.2 (yWorks GmbH, Tuebingen, Germany, www.yworks.com (accessed on 22 January 2022)) software contributed significantly to creating a totally representative sample.

All original Hortobágy HG cow families (cows from the same matrilineal founder) that survived the bottleneck and still exist have been identified and sampled (*n* = 110).

The blood samples were collected from 2020 to 2022 from the Hortobágy herd, Hungary. Hortobágy has for centuries been the main traditional grey cattle breeding center in the Great Plain in Eastern Hungary. They came from the samples annually taken during routine veterinary procedures to detect leucosis, tuberculosis, brucellosis, and IBR. The further use of samples obtained during clinical veterinary procedures for research is not considered an animal experiment under EU Directive 63/2010; therefore, ethical approval is not required.

According to the manufacturer’s instructions, DNA was isolated using the GenElute Blood Genomic DNA Kit (Sigma Aldrich, St. Louis, MO, USA).

The mtDNA control region (CR) comprised between nucleotide positions (nps) 15.718 and 517 was amplified through a Polymerase Chain Reaction (PCR) using the forward and reverse primers 5′-CCTAAGACTCAAGGAAGAAACTGC-3′ and 5′-AACCTAGAGGGCATTCTCACTG-3′ [16]. The 25 μL PCR mixture prepared for each sample contained 2.5 μL dNTP (10 mM), 2.5 μL 10× PCR buffer, 1.5 μL MgCl_2_ (25 mM), two μL primer (10 μM), 1 μL BSA (20 mg/mL), 0.4 μL Taq polymerase (5 U/μL) (ThermoFisher Scientific, Waltham, MA, USA), a 10 ng DNA template, and PCR-grade water to volume. The 1138 base pair (bp) PCR fragments were sequenced using the BigDye^®^ Terminator version 3.1 Cycle Sequencing Kit (Thermo Fisher Scientific, Waltham, MA, USA) following the manufacturer’s protocol. An ABI Prism 3130XL Genetic Analyzer (Applied Biosystems, Foster City, CA, USA) was used for sequence detection. Sequence data were aligned to the Bovine Reference Sequence (BRS, GenBank Accession number V00654) [23] and analyzed using SeqMan Ultra 17 (DNASTAR, Inc., Madison, WI, USA) software. Mitochondrial sequences were recorded in GenBank with accession numbers PQ572265–PQ572374.

Genetic diversity indices, such as haplotype and nucleotide diversity, were estimated with DnaSP 6 software, a bioinformatics software package that performs extensive population genetics analyses from DNA sequence data [24]. The phylogeny of the HG was inferred by a Maximum Parsimony (MP) approach using the mtPhyl v 5.003 software tool, specifically designed for human mtDNA analysis and phylogeny reconstruction [25], but using an adapted version for the bovine mitogenome analysis. The resulting MP tree was then manually verified and adjusted for the maximum parsimony analysis, as described elsewhere even for other animal species [26,27,28].

## 3. Results

### 3.1. Founder Sampling

The identification of the surviving Hortobágy cow families was carried out on the basis of the electronic herd book. The electronic herd book of the Hungarian Grey Cattle Breeders Association (Magyar Szürke Szarvasmarhát Tenyésztők Egyesülete, MSZTE) contains data on the breed from all available breeding documents after World War II. The cow families were drawn from the Hortobágy cow database using the visualization software yED v3.23.2. From there, the remaining 110 cow families were identified from the bottleneck population in 1962. The animals for the study sample were selected from the surviving cow families. Afterward, 500 μL of whole-blood samples were obtained from the National Food Chain Safety Office sample bank (–20 °C), sent by the Hungarian Grey Cattle Breeders Association for parentage testing from Hortobágy in 2022–2023.

### 3.2. Mitochondrial Control Region Sequence Diversity

Total DNA was extracted from the blood samples. After the PCR amplification and sequencing of the mtDNA control region, electropherograms were aligned and compared to the complete mtDNA Bovine Reference Sequence (BRS, GenBank V00654) [23] in order to identify and register any mutational differences from np 15,764 to np 201, thus resulting in 777 bp long sequences (Table S1).

The total number of trimmed nucleotide sites and sequences was 771 nucleotides (from 15.764 to 201 on the BRS), with a sample size of *n* = 110. The total number of sites, excluding those with gaps and/or missing data, was 759. There were 702 invariable (monomorphic) sites and 57 variable (polymorphic) sites. The latter meets the total number of mutations as well.

There were 16 singleton sites and 41 parsimony informative sites. The number of haplotypes in the sample was 47. The haplotype diversity (Hd) was 0.941, while the standard deviation (SD) of the haplotype diversity was 0.016. The nucleotide diversity (π) was 0.00482, with a standard deviation of 0.00033. The nucleotide diversity, as demonstrated by the Jukes and Cantor method (π-JC) was 0.00484. The average number of nucleotide differences (k) was 3.661. The pairwise difference diagram of the mismatch distribution of the CR sequences is unimodal.

### 3.3. Mitochondrial DNA Haplotypes and Phylogeny

**Figure 1:**
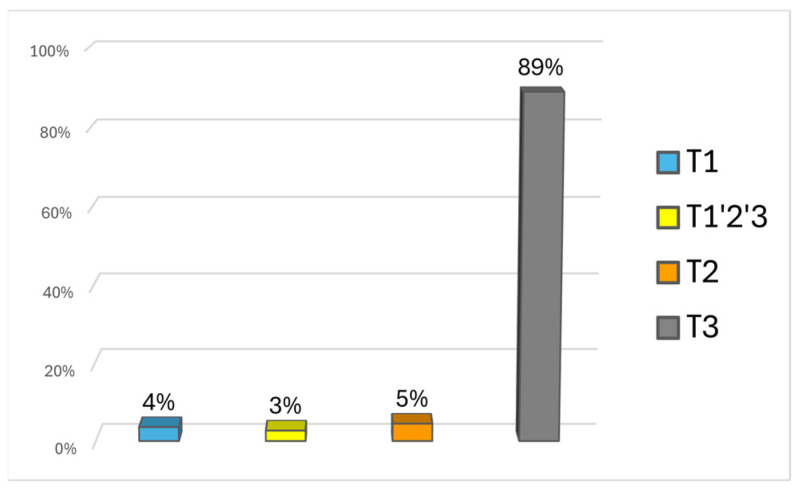
A bar chart showing sub-haplogroup frequency based on the mtDNA control region sequencing of the Hungarian Grey cattle dataset with complete representation.

The analysis of mutational motifs identified so far [29] allowed the classification of all our haplotypes within the T haplogroup. Specifically, samples were classified into the T1, T2, T1′2′3, and T3 haplogroups (Figure 1 and Table S1). As known, for almost all the cattle breeds from the European and Mediterranean areas, the haplogroup T3 resulted as the most common and widespread among all breeds, with the only exception being the Northern African region, where the T1 haplogroup is predominant [30,31,32].

In the totally represented HG sample, only haplotypes within T sub-haplogroups, typical of taurine cattle, were found at the mitochondrial CR level. The predominant haplogroup found in HG cattle was T3 (*n* = 98), followed by T2 (*n* = 5) and T1 (*n* = 4) (Figure 1 and Table S1). Three samples were classified as T1′2′3 (*n* = 3) due to the only mutation in np 16,255 of the mtDNA control region thus showing an apical position in the tree.

The total-representation sample of the HG here tested does not contain ancient rare haplogroups occupying earlier diverging branches of the taurine mtDNA phylogeny, probably derived from European aurochs [17,30].

Considering all of the samples, which numbered 110, the total representation consisted of 89.1% of haplogroup T3, 4.5% of haplogroup T2, 3.6% of haplogroup T1, and 2.7% of haplogroup T1′2′3.

**Figure 2:**
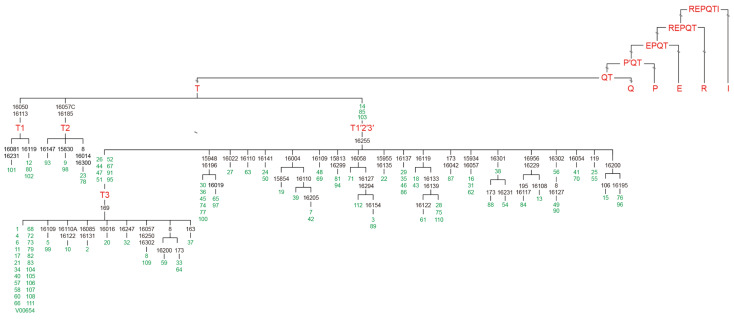
A schematic representation of the HG mtDNAs within the *Bos taurus* phylogeny. This tree encompasses 110 sequences marked with their original IDs (further information is reported in Table S1) all falling within the T haplogroup. The represented topology is one of the most parsimonious trees, differing mainly in the different reconstruction of mutations at highly recurrent sites. Branches display mutations with nucleotide positions according to the BRS (V00654); they are transitions unless a base is explicitly indicated for transversions. Haplogroups are in red and the original sample IDs are in green.

The phylogenetic tree reconstruction shown in Figure 2 graphically reports the genetic relationship within the HG—24 out of 110 samples matched with BRS and sub-branches within T3 are detectable, but not sufficiently informative for a sub-haplogroup classification due to the partial mitogenome sequence tested in this study (Figure 2).

Clades attributable to the T1 and T2 haplogroups revealed, respectively, two and three haplotypes with one unique haplotype in both haplogroups. Twenty-two haplotypes within T3 were unique, thus reflecting the accuracy of sampling focused on excluding the maternally closer lineages.

## 4. Discussion

Recently, many efforts have been made to verify line and family assignations based on pedigree data in livestock, especially in horse breeds. MtDNA sequencing has been extensively used as a candidate molecular marker to verify the accuracy or uncover possible assignment errors [33,34,35,36,37] and to provide genetic insights in mating programs to preserve historical pedigrees [33,35,38,39,40,41].

Only in the last few years has this methodological approach been applied in cattle. Brajković and colleagues [42] promoted the use of mitogenome information for genealogical verification with the identification of pedigree errors. Fortuna and colleagues [43] determined the effect of mDNA in dairy breeding practices and accounted through simulations how mDNA improves genetic evaluations and genetic gain.

From a mitochondrial DNA point of view, as expected, the mtDNA haplotypes were not preferentially distributed among breeds, thus confirming the impossibility of employing the mitochondrial genome as a cattle breed-specific marker.

Within all the available HG sequences, our results showed a high genetic variability demonstrated by a high haplotype diversity index (Hd = 0.94), especially if compared to other Podolian breeds grouped together, thus supporting previous research results; in any case, the HG Hd was comparable to those found in Maremmana, Marchigiana, Romagnola, and Piedmontese [2]. This can be attributed to their relatively shorter history of artificial selection. Among those breeds, the haplogroup composition of the HG perfectly mirrors only the Maremmana breed. Thus, it was included in the analysis, even to test if past crossbreeding is still evident in extant herds.

The HG is a taurine Podolian beef cattle breed phylogenetically conserved in its original state. This is because it has not been subjected to intensive crossbreeding practices with other breeds for changing breeding purposes (dual-purpose or dairy). The original breeding goal of this beef cattle breed has been preserved for centuries and, as we know, the breeding goal is crucial in animal breeding.

As for the T1 haplogroup, this was found in four out of 110 samples (3.6%), here considered as the total-representation of the HG breed (3% if all Hungarian Grey sequences recorded in GenBank to date are considered). Because of its geographic distribution, haplogroup T1 has been deeply investigated, pointing to a possible origin in the Near East, followed by an independent domestication event in Africa.

**Figure 3:**
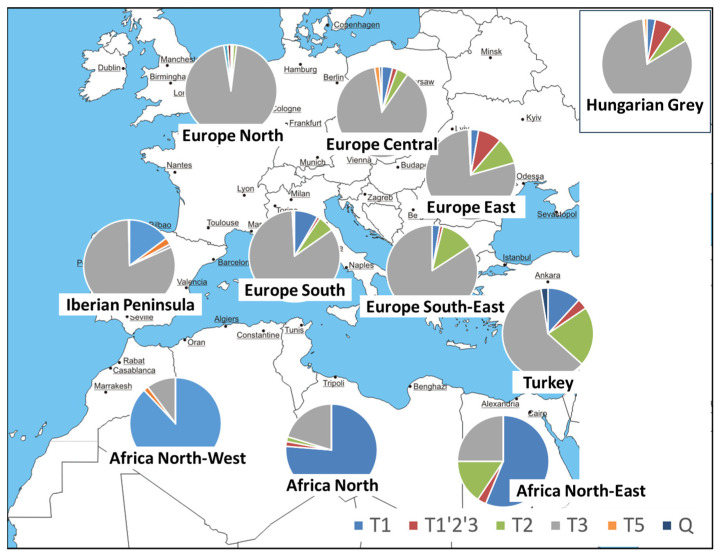
*Bos taurus* haplogroup distribution grouped in macro-geographic areas of Europe, North Africa, and Turkey compared to the HG as reported in the upper box (the map was retrieved from https://d-maps.com/, accessed on 11 November 2024).

Haplogroup T1 is prevalent in African breeds with a frequency of 77%, with peaks of 90% in Tunisia and Morocco (Figure 3 and Table S2 for details).

T1 has also been reported in Turkey (11%) and some of the South European breeds, particularly the Iberian Peninsula, show a higher degree of African influence (15%), potentially due to millennia of cattle interactions between North Africa and Europe across the Gibraltar Strait [2]. Also, breeds from the Italian Peninsula showed the T1 haplogroup, especially within the Podolian cattle group, such as Modicana, Italian Podolian, and Maremmana (to mention just a few of them); except for the Agerolese and Cinisara breeds (the only breeds with a T1 frequency >10% not to be considered Podolian [2]). T1 was also found in the Iskar cattle (7%), that are considered a Podolian–Illyrian breed separate from the Bulgarian Grey (https://www.fao.org/dad-is, accessed on 11 November 2024). In addition, Xuan and colleagues (2010) [44] recorded a high T1 frequency in the Romanian Black Spotted breed, but it is not considered here due to its very low sample size (one out of three individuals).

Bonfiglio et al., 2012 [30], when dissecting the T1 phylogenetic tree based on complete mitogenomes and analyzing all control region GenBank entries, supported the hypothesis that at least 7–8 independent female lineages belonging to haplogroup T1 underwent domestication in the Near East and spread across different areas of the world following human migrations.

The haplogroup distribution, in accordance with the dual maternal origin of Podolian cattle presented in Di Lorenzo et al., 2018 [2], is characterized by a great wave of cattle migration that occurred during the V century invasions through an inland route (potentially marked by the T2 haplogroup) and the intensive migrations across the Mediterranean Sea, probably marked by the T1 haplogroup. What is more, all HGs belonging to the T1 and T2 haplogroups presented unique haplotypes, never detected in taurine cattle before (see Table S2), harboring globally private mutations, only shared at most within the HG. The importance of further investigations of those specific lineages, by performing a complete mitogenome sequencing to be able to deepen the HG phylogeny, estimate their divergence times, and hypothesize their origin, is undeniable.

T2 is quite common in Europe and the Middle East, especially in some Italian and East European breeds (Figure 3). Complete T2 mitogenomes have also been recorded in the Balkans, Egypt, and the Near East, but they were all different from the HG haplotypes, thus enhancing the need for further comprehensive investigations.

Beja-Pereira first published the HG CR haplotypes in 2006 [14]. The HG sequences (*n* = 12) marked as belonging to the article were downloaded from GenBank (DQ515389–DQ515400) and contain exclusively T haplotype groups, although in a different proportion than shown in the figure in the article. The published T3 proportion shown in the figure is higher than in the uploaded sequences (published 80%, GenBank 50%). However, despite the lack of data on the sample design, overall, their results match the haplogroups in this research.

The Croatian indigenous Podolian breeds Busha, Istrian, and Slavonian Syrmian Podolian were tested for mtDNA CR [45]. In the study, it was declared that the samples were derived from unrelated animals (*n* = 146). The CR haplotype groups were predominantly in T3, with few in T2 and a negligible number in T5. Here, the results are used in gene conservation because, similarly to the Hungarian Grey breed’s history, there was a very strong bottleneck effect in the breed after the Second World War, and mitochondrial diversity is also considered important.

Ilie’s paper investigated the STR and mitochondrial CR stages of Romanian greys [46]. The aim here was to support the gene conservation work of the breed in a critical status. The sample design method is declared random and non-related without mentioning the pedigree. Mitochondrial CR sequencing identified the T3, T2, and T1 haplogroups.

Mitochondrial CR haplogroups for Podolian breeds were reported by Di Lorenzo in 2018 on the dual maternal origin of Podolian cattle breeds [2]. For the presumed distinct Podolian breeds, the first subgroup included Italian breeds with Turkish Grey, while the second subgroup included all other European breeds. The two groups were based on geography. The method of designing the test samples was not declared. The sequencing methodology used in the publication was the same as that used in our study. The results of this study are summarized in a very comprehensive table. The grouping of Podolian breeds is perfectly delineated between the breeds containing aurochs mtDNA haplogroups (R, E, P, and Q) and those containing only T lineages, and the literature investigating the utility of nuclear recombinant STR markers in archaeological samples is cited in the conclusions.

Overall, the extant genetic variability here detected seems to reflect the most significant crossbreeding practice that occurred in the 19th century, when the breed faced competition from modern European breeds like the Holstein, Simmental, and Shorthorn. More interestingly, specific mtDNA lineages (*n* = 4) shared with the Maremmana can testify shipments of Maremmana breeders for crossings with the Hungarian Grey also known to have occurred in the 1930s [47].

The breed histories should also be examined from the point of view of whether the breeding purpose of the breed changed after the development of pure breeding (late 18th century, Colling brothers). If they had been trying to transform and crossbreed the breed to dual purpose or dairy, then the mitochondrial genetic background of the breed would have changed! No such changes were made in the HG, because the first major wave of crossbreeding with Simmentals separated the Hungarian spotted from the Hungarian Grey [48], which could be used much more effectively for milk production.

Davidescu investigated the Romanian grey’s CR in 2022 [21]. The sample design was not declared; the animals came from a gene reservation farm in northern Moldova. Among the 32 samples, 62% belonged to the T3/T4 lineages, 19% to the T2, 16% to the PQT, and 3% to the T1. Within the bovine phylogeny, the PQT haplotype group, indicative of ancestral hybridization, is present in many crossbred Podolian breeds.

These results are presumed to be definitive for the HG due to the complete representation of the breed. The bottleneck population drastically reduced the genetic diversity of the genome in this variety. Due to the specific nature of its inheritance, this impact on mitochondrial DNA diversity cannot be estimated only by examining the pre-bottleneck ancient DNA samples.

Specific breeding options can counteract the effect of genetic erosion. For example, the breeding of Hungarian Grey calves was born in 2021 by In Vitro Fertilization (IVF) from semen frozen in the 1970s.

## 5. Conclusions

The study consists of a total-representation approach aiming to assess the phylogenetic placement of the Hungarian Grey cattle breed within the context of other European breeds. The MtDNA control region sequences describe the maternal genetic structure of the breed and enhance our understanding of cattle’s origin patterns across Eastern Europe.

The present study focused on a total-sample representation and revealed that the HG contains only taurine haplogroups, predominantly T3, T1′2′3, T1, and T2. The original breeding purpose of the Podolian cattle was for beef production. In the original Podolian breeds, this has been preserved and not changed, so they have not been crossed with cattle carrying mitochondrial aurochs haplogroups. On this basis, the Podolian cattle can be divided into two groups: the original ‘primitive’ Podolian bovines bred exclusively as extensive beef cattle and the crossbred Podolian cattle, which were crossbred with highly productive breeds as intensive beef, dual-purpose, or dairy cattle.

The HG has been selectively bred for centuries, primarily for beef production, without significant crossbreeding with high-yield dairy or dual-purpose breeds. The breeding goal was constant. This historical breeding isolation is reflected in the mtDNA results, which indicate a lack of introgression from breeds carrying aurochs-derived haplogroups and the retainment of their original genetic structure. This peculiarity reinforces the importance of targeted breeding programs that avoid dilution with high-production breeds. Furthermore, the genetic uniqueness of certain haplotypes suggests that additional sequencing efforts (such as of complete mitogenomes) could provide deeper insights into the breed’s historical migrations and domestication patterns.

## Data Availability

The original data presented in this study are openly available in GenBank with the accession numbers PQ572265–PQ572374.

## Supplementary Materials

The following supporting information can be downloaded at: https://www.mdpi.com/article/10.3390/ani15091186/s1, Table S1: List of samples analyzed in this study; Table S2: Sources and haplogroup affiliation for the *Bos taurus* control region mtDNA sequences retrieved from GenBank and grouped in macro-geographic areas (www.ncbi.nlm.nih.gov/genbank/ accessed on 11 November 2024), e.g., [49,50,51,52,53,54,55,56,57,58,59,60,61].
